# Altered blood flow due to larger aortic diameters in patients with transcatheter heart valve thrombosis

**DOI:** 10.1063/5.0170583

**Published:** 2023-12-19

**Authors:** Silje Ekroll Jahren, Caglayan Demirel, Karoline-Marie Bornemann, Pascal Corso, Stefan Stortecky, Dominik Obrist

**Affiliations:** 1 ARTORG Center for Biomedical Engineering Research, University of Bern, Bern, Switzerland; 2 Department of Cardiology, Inselspital, Bern University Hospital, University of Bern, Bern, Switzerland

## Abstract

The etiology of transcatheter heart valve thrombosis (THVT) and the relevance of the aortic root geometry on the occurrence of THVT are largely unknown. The first aim of this pilot study is to identify differences in aortic root geometry between THVT patients and patients without THVT after transcatheter aortic valve implantation (TAVI). Second, we aim to investigate how the observed difference in aortic diameters affects the aortic flow using idealized computational geometric models. Aortic dimension was assessed using pre-TAVI multi-detector computed tomography scans of eight patients with clinical apparent THVT and 16 unaffected patients (two for each THVT patient with same valve type and size) from the Bern-TAVI registry. Among patients with THVT the right coronary artery height was lower (−40%), and sinotubular junction (STJ) and ascending aorta (AAo) diameters tended to be larger (9% and 14%, respectively) compared to the unaffected patients. Fluid–structure interaction (FSI) in two idealized aortic models with the observed differences in STJ and AAo diameter showed higher backflow rate at the STJ (+16%), lower velocity magnitudes in the sinus (−5%), and higher systolic turbulent dissipation rate in the AAo (+8%) in the model with larger STJ and AAo diameters. This pilot study suggests a direct effect of the aortic dimensions on clinically apparent THVT. The FSI study indicates that larger STJ and AAo diameters potentially favor thrombus formation by increased backflow rate and reduced wash-out efficiency of the sinus. The reported observations require clinical validation but could potentially help identifying patients at risk for THVT.

## I. INTRODUCTION

The incidence of clinically apparent transcatheter heart valve thrombosis (THVT) is low (<3%),^1,2^ but may affect prosthetic valve function (e.g., increased transvalvular pressure gradient) and cause systemic thromboembolism. Subclinical THVT, however, is frequently found (incidence 7%–35%) using high resolution imaging of the prosthetic valve during routine clinical follow up.^2,3^ THVT is predominantly found on the transcatheter heart valve (THV) leaflet corresponding to the right coronary cusp (RCC) and non-coronary cusps (NCCs),^4,5^ and is associated with smaller prosthetic valve size (<23 mm).^1^ THVT either presents as reduced leaflet mobility (HAM—hypoattenuation affecting motion) or as hypoattenuated leaflet thickening (HALT) due to a thin layer of thrombus on the aortic side of the leaflets. The impact of subclinical THVT on clinical outcomes is largely unknown. Factors linked to subclinical THVT include female sex, older age, small aortic annulus, small height and diameter of the sinus of Valsalva, total valvular calcium volume, lower preprocedural mean aortic valve gradient and peak aortic blood flow velocity.^3,6^

Many factors might lead to THVT including pathophysiological mechanisms, device variables (e.g., valve design, valve size, valve materials, implantation techniques, valve-in-valve implantation), patient variables (e.g., transvalvular pressure gradient, valve calcification, aortic root dimensions and morphology, body-mass-index), and choice of antithrombotic therapy.^2^ Disturbed blood flow past the aortic valve has also been linked to thrombus formation.^7^ Regions of flow with high shear-stress levels promote platelet activation. In combination with flow recirculation zones and regions of low flow or blood stasis, this may lead to platelet adhesion and thrombosis. The configuration of such regions of high stress, recirculation, low flow and stasis depends on aortic root morphology and dimensions, as well as on valve types.^8–13^ Also the positioning of the THV within the aortic root and the location of the coronary ostia influences these flow patterns and the wash-out efficiency of the sinus portions and neo-sinus of THVs.^14–23^

The aortic root morphology and dimensions are patient-specific and sex dependent^24–28^ and are critical for THV device development and proper device selection to avoid complications. Aortic dimensions including ascending aortic dimension, annulus size, sinus dimensions and coronary artery heights are evaluated pre-transcatheter aortic valve implantation (TAVI) for device selection during patient screening.^29^ Additionally, the implantation of a THV has been shown to modify the post-TAVI aortic dimensions.^30^ In our previous *in vitro* study,^11^ we found that the aortic root morphology affects the blood flow in the aortic root and that these effects were also present *in vivo* in patients with a THV. Disturbed blood flow in the aortic root might promote thrombus formation. However, the role of the aortic root morphology in THVT remains unknown, as well as the mechanisms by which differences in morphology affect the risk of THVT.^31^ In the current pilot study, we compared the aortic root morphology in a small sample of patients receiving TAVI with clinical THVT to unaffected TAVI patients. The first aim was to identify in a pilot study on patient data some morphological features which might be connected to the formation of thrombosis. This indicated that larger aortic diameters may play a role in THVT. Second, we aimed to investigate by a computational study the effect of different aortic diameters on the blood flow using two idealized (generic) aortic models. To focus on effects of morphology alone, other well-established confounding factors for THVT (e.g., neo-sinus, THV type and position) were excluded from the studied computational configurations. Based on our previous studies, we hypothesize that certain aortic root morphologies might promote THVT by affecting the high shear stress regions downstream of the THV as well as the wash-out efficiency of the sinus.

## II. RESULTS

### A. Aortic root dimensions—registry data

The selected patients were implanted with five different THV types, and three different THV sizes (23, 26, and 27 mm). The eight THVT patients had an age of 79.0 ± 7.1 years, and the 16 unaffected patients had an age of 84.3 ± 5.6 years. Table I gives an overview of all studied patients, as well as the implanted THV types and sizes.

**TABLE I.** Overview of THVT patients and controls and the implanted THVs. THVT, transcatheter heart valve thrombosis; THV, transcatheter heart valve.

THVT patient	Control patient	THV device	Valve size (mm)
1	9	Medtronic CoreValve	23
	10		
2	11	Edwards Sapien XT	26
	12		
3	13		
	14		
4	15	BSC Lotus	27
	16		
5	17		
	18		
6	19	Symetis Accurate	23
	20		
7	21	Edwards Sapien 3	23
	22		
8	23		
	24		

**FIG. 1.**
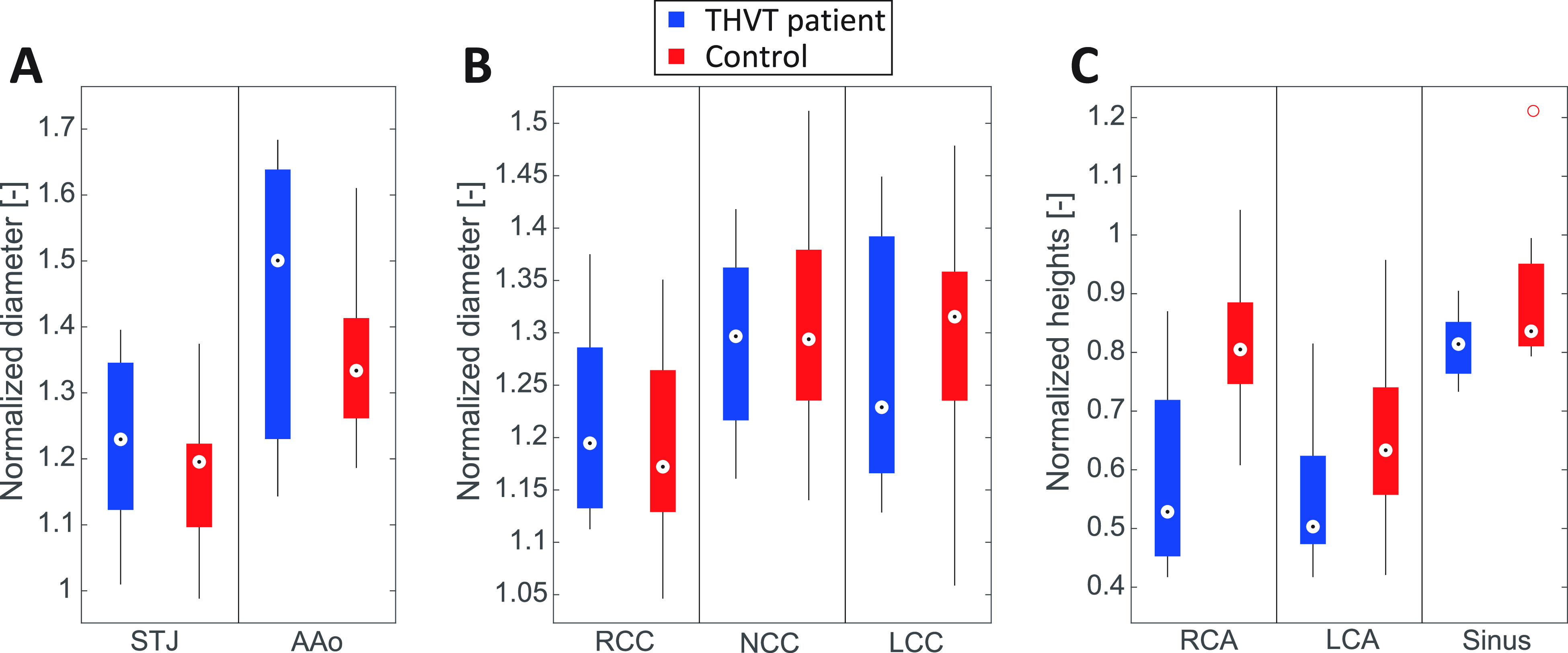
Normalized aortic dimensions (normalized to the annulus diameter) seen in the THVT patients (blue) and the controls (red) pooled for all THVT patients and all control patients, respectively. The bottom and top edge of the boxes indicate the 25th and 75th percentiles, respectively. The median is marked with a black dot within a white circle. The red circle symbol indicates outliers. Normalized diameters of (a) the sinotubular junction (STJ) and the ascending aorta (AAo), and (b) the sinus portions corresponding to the right coronary cusp (RCC), non-coronary cusp (NCC) and the left coronary cusp (LCC). (c) Normalized heights (distance to annulus) of the right coronary artery (RCA) and the left coronary artery (LCA) and the normalized height of the sinus portion (distance between annulus and STJ).

Figure 1 shows all the normalized aortic root dimensions of all THVT patients and all controls. The medians of the ascending aorta (AAo) and sinotubular junction (STJ) diameters [Fig. 1(a)] in the THVT patients are larger than in the controls, but not statistically significant. The sinus diameters [Fig. 1(b)] are not significantly different compared to control (p > 0.05). The medians of the right coronary artery (RCA), left coronary artery (LCA), and sinus height [Fig. 1(c)] in the THVT patients are lower than in the controls. The difference is statistically significant for the RCA height (p < 0.05). Table II gives an overview of the average aortic root dimensions in all THVT patients and all patients in the non-THVT group, which indicates small differences between the two groups.

**TABLE II.** Average aortic dimensions and standard deviations measured in the THVT patient and the control patients. STJ, sinotubular junction; AAo, ascending aorta; RCC, right coronary cusp; LCC, left coronary cusp; NCC, non-coronary cusp; RCA, right coronary artery; LCA, left coronary artery; THVT, transcatheter heart valve thrombosis.

Dimension	THVT patient (mm)	Control patient (mm)	THVT patient normalized to annulus (−)	Control patient normalized to annulus (−)
Annulus	23.5 ± 4.1	23.7 ± 2.8	⋯	⋯
STJ	28.3 ± 2.6	27.7 ± 2.7	1.22 ± 0.14	1.18 ± 0.11
AAo	33.2 ± 2.1	31.6 ± 2.0	1.45 ± 0.22	1.35 ± 0.11
RCC sinus	28.3 ± 3.7	28.0 ± 3.5	1.21 ± 0.10	1.19 ± 0.09
LCC sinus	29.5 ± 4.3	30.7 ± 3.9	1.27 ± 0.13	1.30 ± 0.11
NCC sinus	30.0 ± 3.7	30.4 ± 3.6	1.29 ± 0.09	1.31 ± 0.10
Sinus height	19.0 ± 3.2	20.9 ± 2.3	0.81 ± 0.06	0.89 ± 0.11
RCA height	13.4 ± 2.6	19.3 ± 2.7	0.59 ± 0.17	0.82 ± 0.11
LCA height	12.9 ± 3.2	15.2 ± 3.0	0.55 ± 0.13	0.65 ± 0.14

**FIG. 2.**
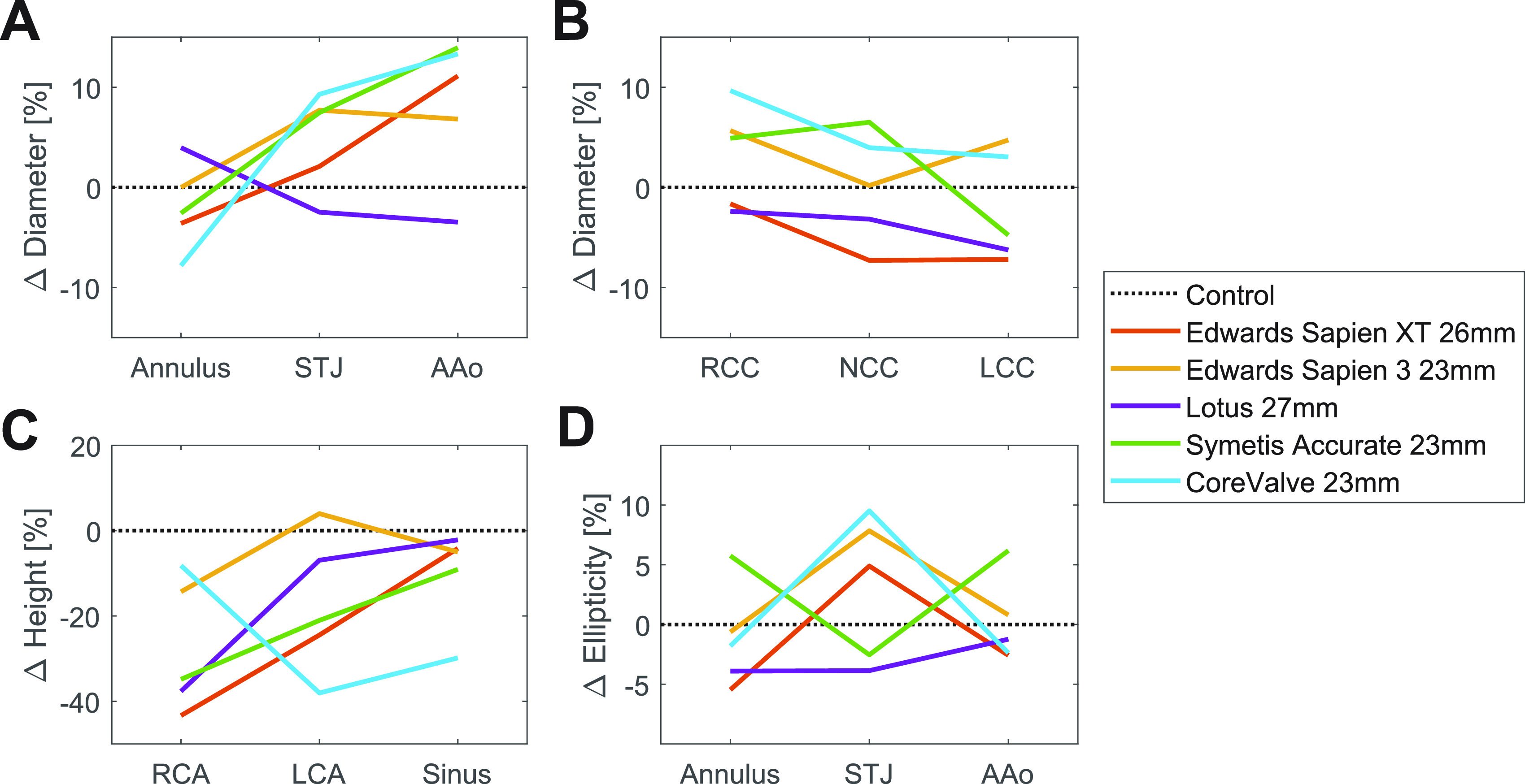
Difference between the normalized dimensions of the THVT patients compared to their specific controls (0%) for each valve type and size for all aortic and sinus dimensions. Calculated as 100. (Patient value − control value)/control value. Difference in (a) aortic diameters, (b) sinus diameters, (c) heights of the coronary arteries (distance from annulus) and the sinus portion (distance between annulus and STJ), and (d) ellipticity of the different aortic cross sections. STJ, sinotubular junction; AAo, ascending aorta; NCC, non-coronary cusp; RCC, right coronary cusp; LCC, left coronary cusp; RCA, right coronary artery; LCA, left coronary artery.

Figure 2 show the relative differences between all normalized aortic root dimensions of the THVT patients and their specific controls for each valve type. The Aao and STJ diameters [Fig. 2(a)] are larger in the THVT patients than in the specific controls, except for the patients with the Lotus 27 mm valve which shows the opposite pattern. This trend is strongest for the AAo diameters which are approximately 10% larger in the THVT patients. For the sinus diameters [Fig. 2(b)] no clear trend can be observed. The RCA and the LCA heights [Fig. 2(c)] are clearly smaller in the THVT patients than in their specific controls (except for the LCA of the Edwards Sapien 3 23 mm valve which was higher). This trend is strongest for the RCA heights which shows differences of approximately 40% for Edwards Sapien XT 26 mm, Lotus 27 mm, and Symetis Accurate 23 mm. The sinus height is smaller in all THVT patients compared to control with 5%–10%. For ellipticity [Fig. 2(d)], no trend in the difference between THVT patients and their specific controls could be observed.

### B. Impact of larger STJ and AAo diameter on systolic aortic root blood flow—Computational study

Two idealized aortic root (generic) models were designed based on the observed differences in the STJ and AAo diameters [Figs. 1(a) and 2(a)]. These models were then used in a computational study of the systolic blood flow. To this end, values close to the median values of the normalized diameters were used. The THVT model diameters were set to $d_{\text{STJ}}=1.25\cdot d_{A}$ and $d_{\text{AAo}}=1.50\cdot d_{A}$, and the control model to $d_{\text{STJ}}=1.15\cdot d_{A}$ and $d_{\text{AAo}}=1.30\cdot d_{A}$ (with $d_{A}=22\;\text{mm}$). The resulting total fluid volumes in the AAo and in the sinus portion of the THVT model [Fig. 7(b)] were, therefore, larger than in the control model (AAo: $2.58\cdot 10^{-5}$ m^3^ vs $1.99\cdot 10^{-5}$ m^3^, Sinus: $0.40\cdot 10^{-5}$ m^3^ vs $0.37\cdot 10^{-5}$ m^3^).

**FIG. 3.**
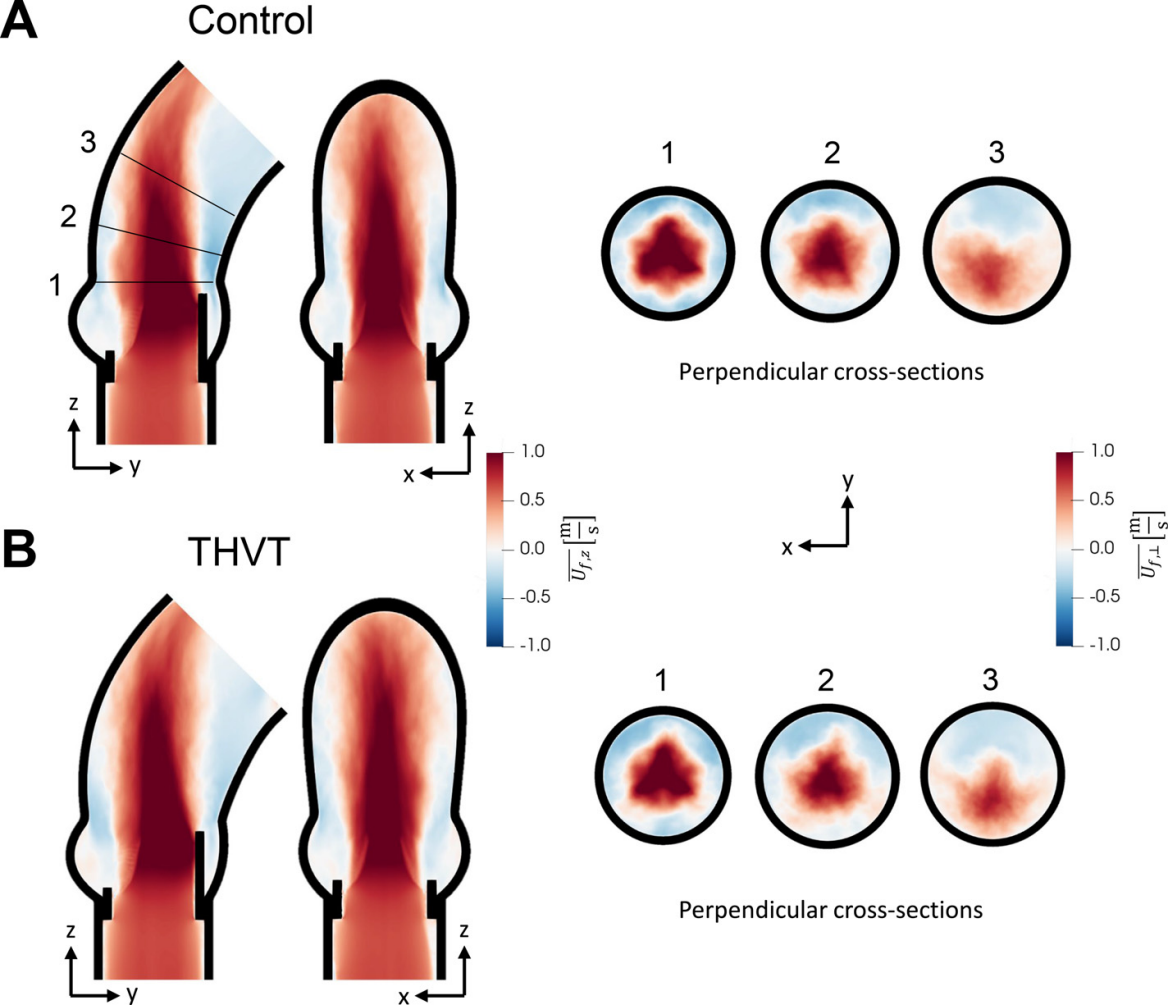
The average velocity field from the computational analysis in the (a) control and (b) the THVT aortic models, seen from two different views normal to the main flow direction, and for three perpendicular cross sections at (1) the sinotubular junction, (2) in the ascending aorta and (3) in the bend toward the aortic arch.

**FIG. 4.**
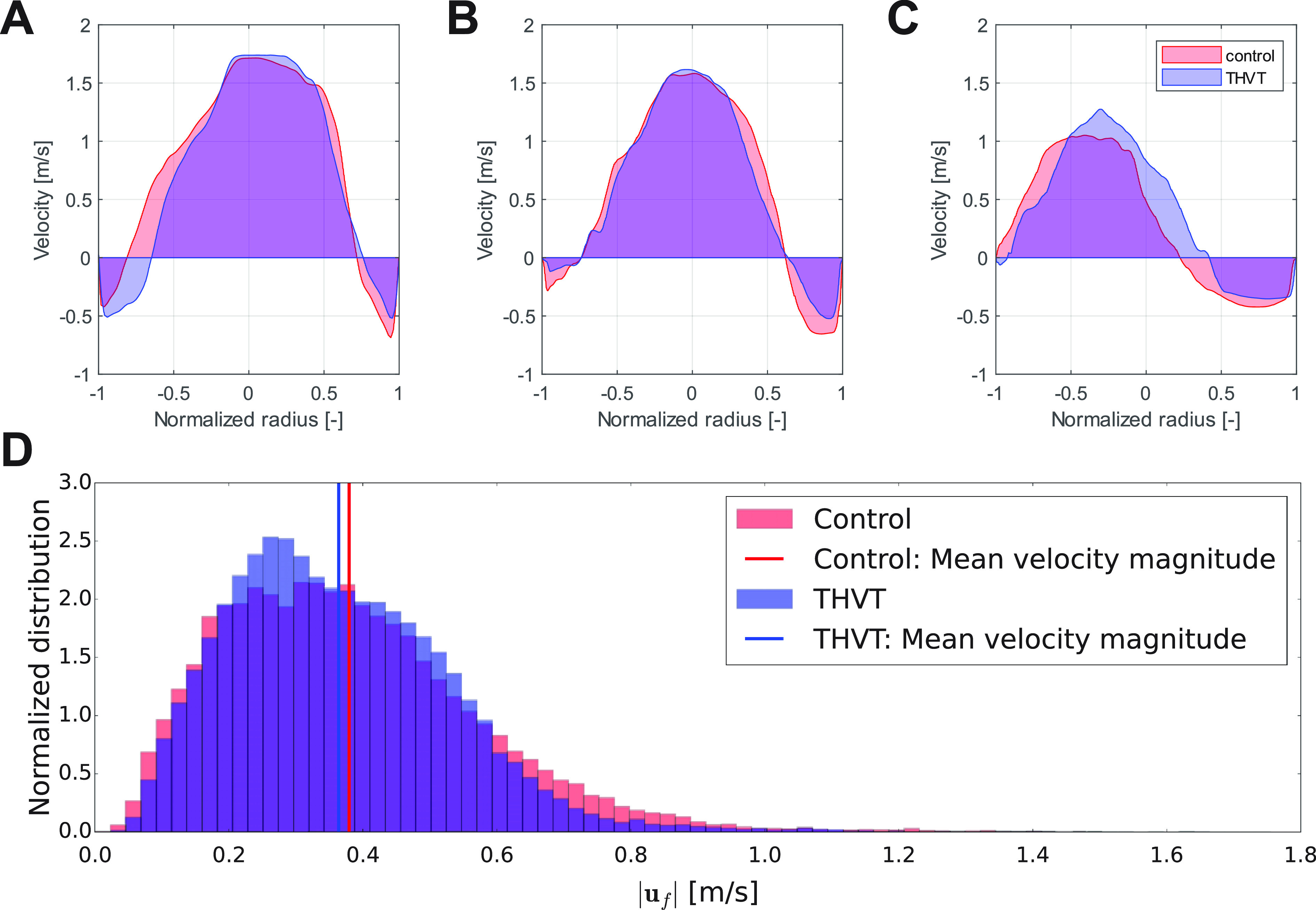
Velocity profiles at three different cross sections in the ascending aorta: (a) plane 1 at the sinotubular junction, (b) plane 2 in the ascending aorta, and (c) plane 3 in the bend toward the aortic arch (see Fig. 3). The flow velocity is taken perpendicular ( $\bar{u_{f,⊥}}$) to the cross-sectional plane. (d) Histogram for distribution of the velocity magnitudes within the sinus portions for the control model and the THVT model. The vertical lines indicate the mean velocity magnitude in the control (red) and THVT (blue) model.

Figure 3 shows the peak systolic mean velocity fields in the two aortic root models. The peak systolic velocity of the central aortic jet was similar in both cases ( 2.00 ± 0.50 m/s in THVT vs 2.05±0.43 m/s in control), but the jet in the AAo of the THVT model was wider than in the control. Figures 4(a)–4(c) shows the velocity profiles at three positions in the AAo (same cross sections as in Fig. 3) in both models. At the STJ [Fig. 4(a), cross section 1], the jets are comparable in width and velocity magnitude. At cross section 2 and 3 [Figs. 4(b) and 4(c)], higher backflow velocities along the aortic wall can be observed for the control model. At cross section 3, the jet is wider and more central in the THVT model compared to the control model.

Table III shows the mean velocities and flow rates at the STJ cross section for the two aortic models. Both models exhibit retrograde flow along the walls of the proximal AAo. The associated backflow velocities in the THVT model are lower than in the control model ( −0.34 m/s vs −0.38 m/s), while the area occupied by backflow (blue areas in the cross sections in Fig. 4) is larger ( 42.1% vs 37.9%). The resulting backflow rate is larger in the THVT model (6.0 l/min vs 5.2 l/min). The results are in line with our previous *in vitro* findings using tomographic particle velocimetry in different sized aortic roots:^10^ we measured similar peak systolic velocities, and it was found that backflow rate increased with larger aortic roots.

**TABLE III.** Temporal-averaged values and standard deviations of the mean velocities and flow rates at the STJ cross section in the control and THVT model in the computational study. THVT, transcatheter heart valve thrombosis; STJ, sinotubular junction.

	Control	THVT
Backflow area per total STJ area (%)	37.9 ± 3.9	42.1 ± 4.6
Mean jet velocity averaged over jet area (m/s)	0.95 ± 0.13	0.93 ± 0.15
Mean backflow velocity averaged over backflow area (m/s)	−0.38 ± 0.05	−0.34 ± 0.03
Backflow flow rate (l/min)	−5.2 ± 1.2	−6.0 ± 1.1

**FIG. 5.**
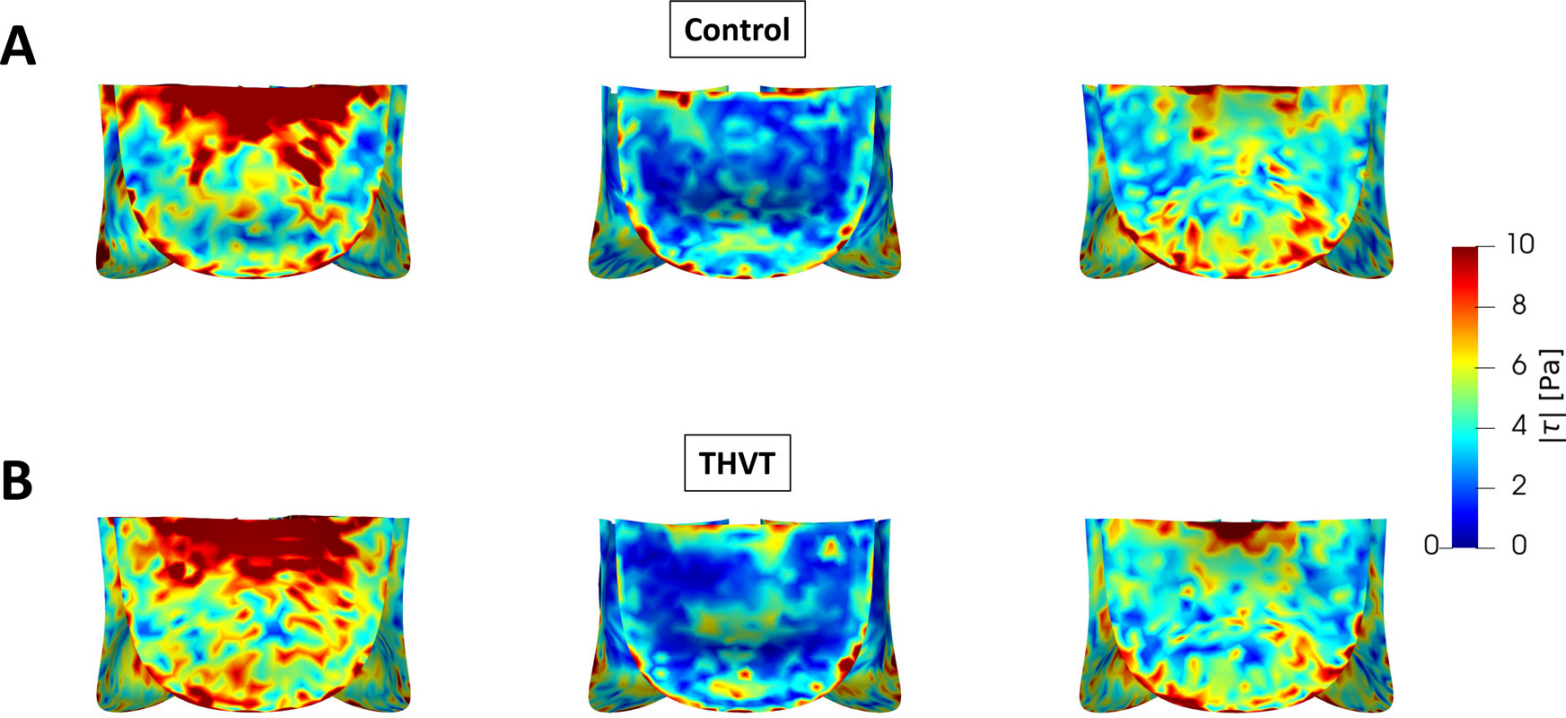
Instantaneous wall shear stress (τ) magnitude on the valve leaflets at three different leaflet positions during systole for the control (a) and THVT model (b).

In the AAo, the total systolic turbulent dissipation rate ( $P_{\text{turb}}$) is higher in the THVT model than in the control model (0.022 W vs 0.020 W), whereas it is lower in the sinus portion (0.00086 W vs 0.00090 W). This difference in the turbulent dissipation rate in the sinus is also reflected in Fig. 4(d) which shows normalized histograms of the systolic velocity magnitude distribution in the sinus portions of both models. The distribution of velocity magnitudes in the control model tends toward higher velocities, while the peak of the THVT model is located at lower velocities. Likewise, the mean velocity magnitude is lower for the THVT model (0.36 m/s for THVT vs 0.38 m/s for control). Figure 5 shows that the systolic instantaneous wall shear stress (WSS) magnitudes on the valve leaflets during systole were similar for both the control and the THVT model.

## III. DISCUSSION

The primary objective of this pilot study was to investigate differences in aortic morphology between a small sample of TAVI patients with clinical THVT and patients who did not develop THVT. Morphological differences if evident may potentially be used to identify patients at risk to develop THVT. The aortic dimensions of eight TAVI patients with THVT were evaluated and compared to 16 unaffected TAVI patients (two per THVT patient with the same specific valve type and size).

Patients with THVT were found to have significantly lower right coronary artery height (up to 43%). Compared to their specific controls (Fig. 2), the sinus height and the annulus diameter tended to be lower (up to 30% and 8%, respectively), and the sinotubular junction (STJ) diameter and ascending aorta diameter tended to be larger (up to 9% and 14%, respectively) in the THVT patients. A small aortic annulus and small sinus height have been linked to subclinical THVT,^6^ similarly a small THV size has been linked to clinical and subclinical THVT.^1,6^ However, the diameters of the STJ and the ascending aorta and the height of the coronary arteries have so far not been associated with THVT. Our idealized computational study showed that a larger STJ and AAo led to a different flow field in the aortic root (Fig. 3). It was associated with lower backflow velocities at the STJ (Table III) and lower velocity magnitudes in the sinus [Fig. 4(d)] as well as a higher systolic turbulent dissipation rate in the AAo and a lower one in the sinus.

The coronary arteries are known to influence the flow field in the sinus portions.^19,20,22^ Moore *et al.*^19^ found that there was more flow in the coronary sinuses along the base of the sinus compared to the non-coronary sinus. This higher flow might influence the wash-out efficiency of the sinus portion and reduce flow stasis. Additionally, coronary flow affected the flow pattern in the sinus by preventing full recirculation and diverting the flow toward the ostia. Hatoum *et al.*^22^ found that the particle residence time in the coronary sinuses was shorter than in the non-coronary sinus. Querzoli *et al.*^20^ observed that the lifetime of the sinus vortex was reduced in the non-coronary sinus. In the present study, the right coronary artery height was found to be significantly lower in patients with THVT compared to the control patients [Fig. 1(c)]. The right coronary artery height is also lower compared to the general TAVI population.^1,24^ The left coronary artery height was also found to be lower when comparing the THVT patients with their specific controls with the same valve type and size [Fig. 2(c)]. These differences in the positions of the ostia are expected to affect the flow field in the sinus portion and possibly also in the neo-sinus after TAVI. The THV leaflet corresponding to the right coronary sinus has been shown to be more affected by thrombus formation^4,5^ together with the leaflet corresponding to the non-coronary sinus. Altered flow fields due to lower ostia might promote thrombus formation if regions of blood stasis are enlarged.

In aortic stenosis treated with TAVI, a dilation of the aortic root is a common finding^26,37^ and has been associated with adverse outcomes after TAVI (higher mortality risk, higher incidence of paravalvular regurgitation, and higher risk of major or life-threatening bleeding). However, the possible link to THVT has not yet been established. In this pilot study, the AAo and STJ diameters were comparable to the general TAVI population,^1,24^ but a clear trend toward larger AAo and STJ diameters could be observed in the THVT patients when they were compared to their specific control patients [Fig. 2(a)]. This might indicate that a larger aortic root promotes the formation of thrombus on the THV. However, statistical significance of this trend could not be tested due to the small sample size. Therefore, these observations should be seen as hypothesis-generating results, and a larger clinical study is necessary to validate the findings.

A secondary objective of the current study was to investigate the relevance of larger AAo and STJ diameters to THVT performing an idealized computational fluid–structure interaction (FSI) study. The flow fields observed in the two idealized models (a THVT and a control model) with the observed differences in STJ and AAo diameter were found to be different (Fig. 3). In the larger AAo (THVT model), the total systolic turbulent dissipation rate was higher (10%) than in the smaller AAo (control model). This difference could be attributed to the larger volume in the THVT model giving more room to turbulent flow. Alternatively, this result could also indicate regions of elevated turbulent dissipation in the THVT model. Either way, the higher total systolic turbulent dissipation rate may promote platelet activation in the turbulent jet distal to the valve in the THVT model. In both aortic models, a backflow along the aortic wall was observed (Table III), which has been linked to the flow and recirculation in the sinus portions.^10,11,20^ This backflow may play two opposing roles in THVT: one role in promoting thrombosis by transporting activated platelets from the turbulent aortic jet to the sinus portions, and another role in attenuating thrombosis by driving the wash-out of the sinus portions. Which role prevails in an actual setting, may be the result of a subtle balance between the two effects which could tip either way. In the THVT model, the flow rate of this backflow was found to be higher (16%) while the backflow velocities were lower (−11%) than in the control model. In addition, the turbulent dissipation rate in the sinus portion of the THVT model was found to be smaller (−4.5%). These observations could indicate that more potentially activated platelets are transported to the sinus due to the higher backflow rate, and that there is less fluid motion in the sinus (due to lower backflow velocities, lower sinus velocities and lower sinus turbulent dissipation rate) in the THVT model resulting in a less efficient washout of the sinus. Accordingly, the wider distribution of velocity magnitudes and the higher mean velocity magnitude within the sinus portion in the control model [Fig. 4(d)] could indicate more intense flow at higher Reynolds numbers in the sinus and, therefore, more efficient wash-out. In summary, the combination of higher turbulent dissipation rate in the AAo, higher backflow rate and less fluid motion in the sinus might explain why patients with larger Aao seems to be more prone to develop THVT.

Limitations: This pilot study is limited by the low number of THVT patients, due to the low incidence rate of THVT detected at routine clinical follow-up, and the limited number of specific controls (two per THVT patient). Therefore, the clinically observed results should be seen as indications and hypothesis-generating observations. This limitation, however, could partially be mitigated by the computational study which indicated that the observed anatomical differences affected the flow fields in a way that could promote thrombus formation. Another limitation is the lack of information on thrombus location and topology in the registry which could have added more information to the study. Further, the AAo diameter was measured at a fixed distance of 40 mm from the annulus which might introduce a bias for this dimension (e.g., due to different sinus heights). This method was used, because it is a clinically accepted measure for the AAo diameter.^24^ Additionally, the post-TAVI aortic morphologies were not available. The implantation of THV is expected to modify the pre-TAVI aortic root dimensions (e.g., dilate). However, the impact of Aao increase on the flow pattern was investigated independently of whether the AAo was increased before or after TAVI. The computational study was limited by the idealized morphology of the aortic models and the lack of coronary arteries. In patients, large variations in morphology are observed. However, using idealized models it is possible to investigate the effects of isolated parameters (in this case a larger aorta) on the systolic flow field parameters. The lack of coronaries and coronary flow in the FSI simulations, is expected to influence the flow pattern in the sinus portion, but it is not expected to fundamentally change the main effects of the larger aorta on the flow field (e.g., increased backflow rate and increased AAo turbulent dissipation rate). Another limitation to the computational study was that a surgical prosthetic valve was used as valve model and that native leaflets and neo-sinuses were lacking. Several studies^8–13^ have shown that valve type influence regions of high stress, recirculation, low flow, and stasis. Therefore, valve type is expected to influence the flow pattern in the sinus portion but is not expected to change the main effects of the larger aorta on the flow field. Therefore, we focused the computational study on the effects of a larger aorta observed in THVT patients. THV-specific aspects (e.g., stent frame, neo-sinus, valve design) were not included to avoid obscuring the results by THV-design related aspects. A simple valve model was used to enable insights on basic mechanisms caused only by the larger aorta, which makes the results more generalizable. For a potential clinical application, however, a patient-specific and valve-specific analysis would be necessary to tailor the treatment.

## IV. CONCLUSION

The increased AAo and STJ diameters and the lower coronary artery heights observed in the small sample of THVT patients suggest that patient-specific aortic root dimensions might play a role in THVT. Both the heights of the coronary as well as the larger aortic diameters are known to influence the flow field in the aortic root, and modified blood flow caused by these aortic dimensions might therefore be an important factor in THVT development. The observed increase in turbulent dissipation rates in the AAo and decrease in velocities in the sinus portions in the idealized computational model with larger STJ and AAo could provide an explanation why a larger aortic diameter might promote thrombus formation. If clinically validated, it might be possible to use these dimensions as markers during preprocedural planning of TAVI and subsequent patient monitoring to identify patients at higher risk for THVT. The aortic root dimensions are routinely assessed during the preprocedural planning using multi-detector computed tomography (MDCT), and an analysis of the coronary heights and ascending aortic diameter of patients to support the selection of the best patient-specific anticoagulation scheme could be feasible. However, a larger clinical study is necessary to validate the morphological findings of this pilot study.

## V. METHODS

### A. Aortic root morphology—Registry data

In this pilot study, morphological data from eight TAVI patients who experienced clinical THVT were compared to data from 16 unaffected TAVI patients (free from THVT) with two patients with the same THV type and size for each THVT patient. The THVT patients were identified in the Bern TAVI registry between May 2013 and May 2015 (ten patients experienced THVT in this period, two were excluded due to lack of sufficient information in the registry for the present investigation) and have been described previously.^1^ The unaffected patients were entered in the Bern-TAVI registry between January 2013 and March 2021. The Bern TAVI registry was approved by the local ethics committee and all participants provided written informed consent prior to inclusion (clinicalTrials.gov Identifier: NCT01368250).

**FIG. 6.**
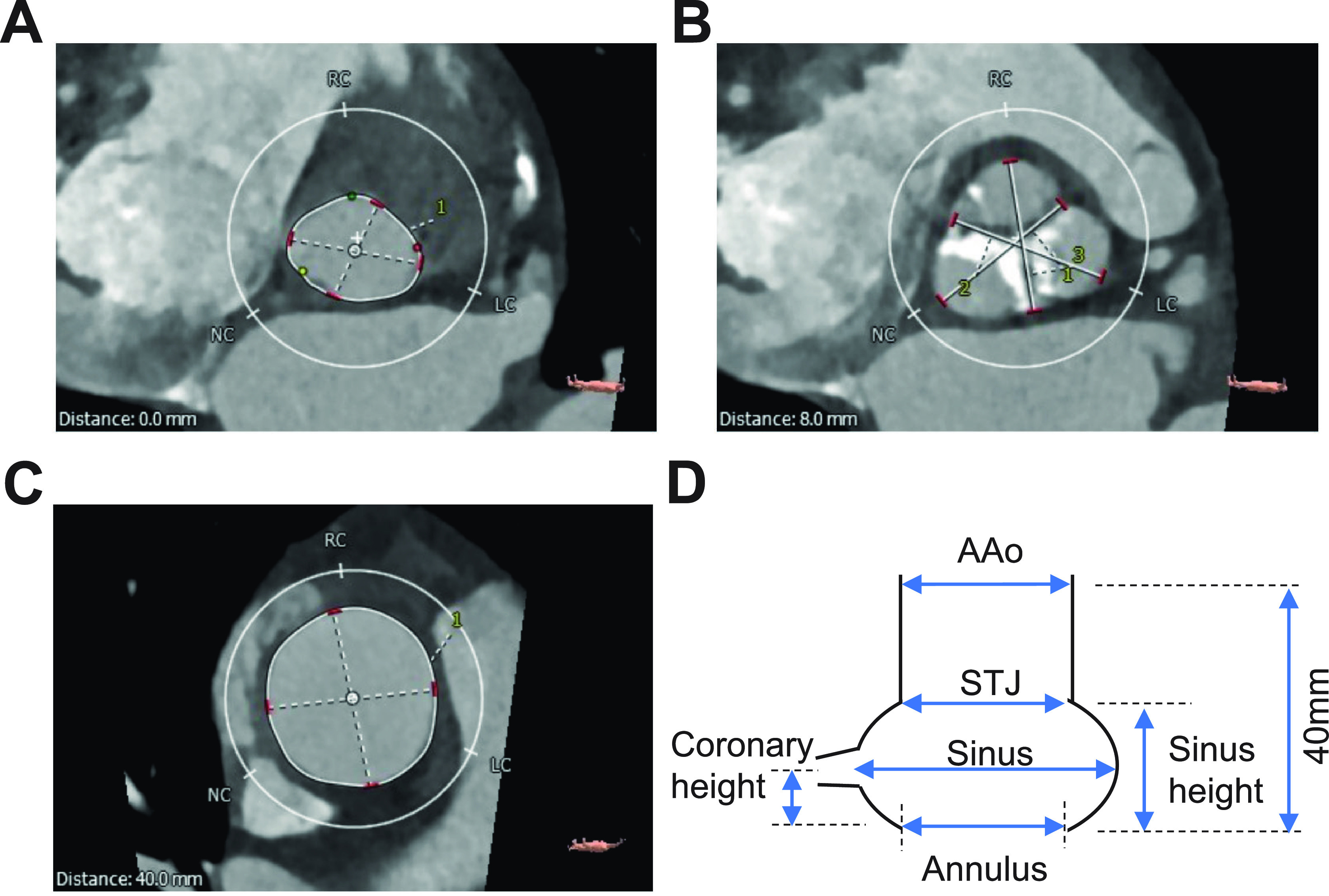
Pre-operative MDCT images of a control patient indicating the measures of maximum and minimum diameter of the different aortic and sinus dimensions. (a) Measures of the annulus diameter (at 0 mm distance from the annulus). (b) Measures of the three sinus portions (at 8 mm distance from the annulus at maximum diameter). (c) Measures of the ascending aorta diameter (at 40 mm distance from the annulus). (d) Schematic of the different measured aortic dimensions. RC, right coronary; NC, non-coronary; LC, left coronary; STJ, sinotubular junction; AAo, ascending aorta.

In each patient, the following dimensions were measured from the pre-TAVI multi-detector computed tomography (MDCT) images: annulus diameter (maximum and minimum), the diameter of each sinus portion corresponding to the non-coronary (NCC), right coronary (RCC) and left coronary cusps (LCC) measured as the largest diameter from the opposing commissure, the height of the right (RCA) and left coronary artery (LCA), the height of the sinus portion [from annulus to sinotubular junction (STJ)], the diameter of the STJ (maximum and minimum), and the diameter of the ascending aorta (AAo) at 40 mm distance from the annulus (maximum and minimum). Figure 6 shows an example of the measured diameters in one of the unaffected patients. Minimum diameter is the smallest diameter, and the maximum diameter is the largest diameter, of the elliptical/circular cross section.

#### 1. Data analysis

The data were analyzed using MATLAB (Mathworks, Natick, MA, USA). In addition to the measured dimensions, the average diameter and the ellipticity (calculated as the ratio of maximum to minimum diameter) of the annulus, the STJ and the AAo were calculated. All dimensions were normalized by the annulus diameter. The average values and standard deviation of the whole THVT patient group and the whole unaffected group (control) were calculated, and the Wilcoxon rank sum test was used to test if the dimensions of the two groups were statistically different (p < 0.05 was considered significant). Additionally, the THVT patients were compared directly to the corresponding cases without THVT (same valve and valve size). Statistical significance was not tested in this case due to limited statistical power of the small sample.

### B. Computational model

**FIG. 7.**
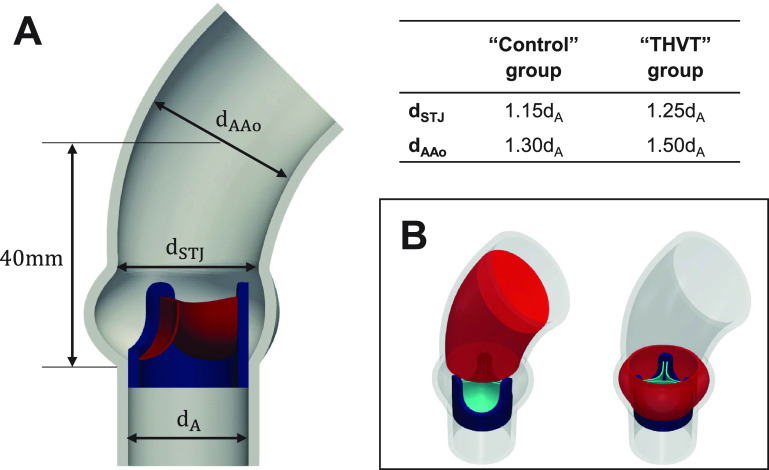
(a) Schematic representation of the parameterized idealized aortic geometry with bioprosthetic valve. The geometry was parameterized relative to the annulus diameter ( $d_{A}=22\;\text{mm}$), and the two aortic dimensions sinotubular junction (STJ) diameter ( $d_{\text{STJ}})$ and ascending aorta (AAo) diameter ( $d_{\text{AAo}})$ were used to create a THVT model and a control model. (b) Inner fluid volumes of the AAo and sinus used to calculate total systolic turbulent dissipation rate.

A computational study was performed to investigate the effects of the identified morphological differences between the THVT patients and the specific unaffected patients (controls) on the peak systolic blood flow patterns in the aortic root. An idealized geometric model of the aortic root was used to generate two different geometrical configurations with different STJ and AAo diameter, selected according to the results from the analysis of the registry data. One geometrical configuration (control case) modeled typical aortic dimensions of the unaffected group and the other configuration (THVT case) modeled the aortic dimensions found in the THVT patient group [Fig. 7(a)]. We chose to study an idealized aortic model to focus on the effect of main geometrical features on the flow field. The average transvalvular pressure gradients seen in the THVT patients (9.4 mm Hg) and general TAVI (7.7 mm Hg) population published in Ref. 1 were imposed in the THVT and control case, respectively.

The unsteady interaction between valve and blood flow was simulated with a high-fidelity solver for cardiovascular fluid–structure interaction (FSI). In this FSI solver, the Navier–Stokes equation governing the flow field were solved by a direct numerical simulation (DNS) approach and discretized with high-order finite differences on a staggered, structured grid with 121×257×513 points. A finite element method for soft tissue was used to solve the full elastodynamics equations for the structure (valve and aorta) on an unstructured mesh with approximately 515 000 tetrahedral elements. The fluid and structural solvers were coupled with a modified immersed boundary method based on variational transfer. The time step is set to $\Delta t=5\cdot 10^{-6}$ s. Further details on numerical implementation and validation can be found in Ref. 32. Pressure boundary conditions are imposed by the fringe region technique. By adding a pressure forcing term to the right-hand side of the Navier–Stokes equations within a fringe region placed at the in- and outflow, a desired transvalvular pressure gradient is achieved.

The fluid was considered Newtonian and incompressible with properties similar to blood (fluid density $\rho_{f}=1050\;\text{kg}/m^{3}$, dynamic viscosity $\mu_{f}=0.004\;\text{Pa·}s$). The aortic wall and valve stent were modeled linearly elastic, valve leaflets were modeled by a fiber-reinforced Holzapfel–Gasser–Ogden (HGO) model.^33^ Parameters in the HGO constitutive equations were chosen according to Ref. 34 and the relative orientation of two sets of fibers was set to 60°. Further technical details can be found in Becsek *et al.*^35^ The geometric model of the inserted bioprosthetic aortic valve is based on the Edwards Intuity Elite 21 valve (Edwards Lifesciences, Irvine, California, USA).

The computational model has previously been validated against *in vitro* experimental data^35^ using tomographic particle image velocimetry of the same valve (Edwards Intuity Elite) and a straight idealized aortic root model with the same dimensions.^10^ The flow pattern of the mean flow and the velocities of the central aortic jet and the backflow were found to agree very well with,^10^ and the results were also consistent with the instantaneous flow field at peak systole in Ref. 36.

For the analysis of the computational results, the temporal mean velocities $\bar{u_{f}\left(x\right)}\;(m/s)$ of the three-dimensional velocity fields $u_{f}=\left[u_{f,x}\;u_{f,y}\;u_{f,z}\right]$ in the fluid domain $x=\left[x\;y\;z\right]$ were calculated by

$$\bar{u_{f}\left(x\right)}=U_{f}(x)=\frac{1}{t_{2}-t_{1}}\int_{t_{1}}^{t_{2}}u_{f}\left(x,T\right)dT$$ (1)

with a temporal averaging interval reaching from $t_{1}=0.2\;s$ to $t_{2}=0.3\;s$ (peak systolic phase).

The strain rate tensor $S\;(s^{-1})$ of the flow field,

$$S=\frac{1}{2}\left[\nabla u_{f}+{\left(\nabla u_{f}\right)}^{T}\right],$$ (2)

was used for calculating the turbulent dissipation rate $\varepsilon \;(m^{2}/s^{3})$ of the flow field,

$$\varepsilon =2\nu_{f}\bar{S^{2}},$$ (3)

with the kinematic viscosity $\nu_{f}=\frac{\mu_{f}}{\rho_{f}}\;(m^{2}/s)$. The total systolic turbulent dissipation rate $P_{\text{turb}}\;(W)$ was calculated by integrating ε over an inner fluid volume V of either the AAo or the sinus portion as [Fig. 7(b)]:

$$P_{\text{turb}}=\rho_{f}\int_{V}^{}\varepsilon dV.$$ (4)

## Data Availability

The data that support the findings of this study are available from the corresponding author upon reasonable request.
